# Prenatal Exposure to Airborne Polycyclic Aromatic Hydrocarbons and Risk of Intrauterine Growth Restriction

**DOI:** 10.1289/ehp.10958

**Published:** 2008-01-30

**Authors:** Hyunok Choi, Virginia Rauh, Robin Garfinkel, Yihsuan Tu, Frederica P. Perera

**Affiliations:** 1 Columbia Center for Children’s Environmental Health, Mailman School of Public Health, Columbia University, New York, New York, USA; 2 Department of Statistics, National Cheng Kung University, Tainan, Taiwan

**Keywords:** air pollution, cephalization index, fetal growth ratio, large for gestational age, polycyclic aromatic hydrocarbons, ponderal index, preterm birth, small for gestational age, symmetric intra-uterine growth restriction

## Abstract

**Background:**

Polycyclic aromatic hydrocarbons (PAHs) are ubiquitous air pollutants generated by combustion of organic material, including fossil fuel.

**Objectives:**

It has been an open question whether prenatal exposure to air pollution in general and PAHs in particular significantly increases the risk of intrauterine growth restriction, including small size for gestational age (SGA), and preterm delivery. Here, we have examined this hypothesis in a cohort of mothers and newborns in New York City.

**Methods:**

Subjects were young, nonsmoking, healthy African-American (*n* = 224) and Dominican (*n* = 392) mother–newborn pairs residing in New York City whose prenatal PAH exposures were estimated by personal air monitoring. Questionnaire and medical record data were obtained.

**Results:**

A 1 natural-log (ln)-unit increase in prenatal PAH exposure was associated with a 2-fold increase in risk of symmetric intrauterine growth restriction (i.e., SGA and fetal growth ratio < 85%) among full-term African Americans (*p* < 0.05). Preterm delivery risk was 5-fold greater among African Americans per ln-unit increase in prenatal PAH exposure. The same unit increase in exposure significantly increased the ratio of head circumference to birth weight by 0.04% in African Americans. These effects were not observed in Dominicans.

**Conclusion:**

Prenatal PAH exposure is likely to contribute to the occurrence of SGA as well as preterm births among African Americans. The lack of an association in Dominicans might reflect modification of the risk by healthful cultural practices among recent Dominican immigrants. Given that PAHs are globally generated and distributed pollutants, our observations have potential implications for environmental health and energy policies.

Polycyclic aromatic hydrocarbons (PAHs) are a class of multiphasic organic compounds, composed of fused aromatic rings (Agency for Toxic Substances and Disease Registry 1995; Bostrom et al. 2002; Finlayson-Pitts and Pitts 1997). They are released into air during incomplete combustion and/or pyrolysis of fossil fuel, industrial or domestic coal, wood, cigarettes, and food items (Bostrom et al. 2002). Growing evidence supports developmental toxicity from prenatal or early postnatal exposure to PAHs (Dejmek et al. 2000; Perera et al. 1998, 2003), in addition to elevated cancer risk (Belpomme et al. 2007) and disruption of the endocrine system (Archibong et al. 2002; Davis et al. 1993). Exposure during the prenatal and early postnatal stages is of particular concern because of the unique susceptibility of the fetus and infant (Huel et al. 1993; National Research Council 1993; Perera et al. 1998, 2004). The prenatal period represents a developmental stage of unique susceptibility, during which exposures can critically affect immune, metabolic, and neurologic functions throughout the life span (Kim 2004; Pinkerton and Joad 2006; Schwartz 2004). This unique vulnerability to xenobiotics may be attributed to the immaturity of developing immune systems, the rapid development of fetal organs, and the fact that exposure per body weight is much greater than that for adults (Pinkerton and Joad 2006; Schwartz 2004).

Transplacental exposure to PAHs released from cigarette smoke induced a large increase in xenobiotic metabolism by the placental tissues, increased PAH–DNA adduct levels, and subsequently reduced neonatal weight in a cohort of mother–newborn pairs (Sanyal et al. 1994). More recent research also supports fetal growth reduction associated with transplacental PAH exposure in humans (Dejmek et al. 2000; Perera et al. 1998, 2003). In a rat model, transplacental exposure to airborne benzo[*a*]pyrene (BaP) has been shown to induce fetal loss and reduce plasma hormones progesterone, estrogen, and prolactin in a dose-responsive manner (Archibong et al. 2002). Exposure to BaP and 7,12-dimethyl-benz[*a*]anthracene (DMBA) during organogenesis leads to significant reduction in birth weight (BW), crown–rump length, and placental proficiency as measured by protein weight and diameter (Sanyal and Li 2007). Fetal cranium and neural tissues appear to be particularly sensitive to BaP and DMBA exposure (Sanyal and Li 2007). For example, transplacental exposure to BaP significantly depressed levels of *N*-methyl d-aspartate receptor subunit 1 within the hippocampus and, after birth, impaired long-term potentiation, a marker of long-term memory and learning (Wormley et al. 2004).

In humans, the risk associated with urban ambient PAH exposure and, more generally, air pollution for intrauterine growth restriction (IUGR) and preterm delivery has remained unclear (Šrám et al. 2005). Epidemiologic studies using area-level air pollutant exposure monitoring data and individual-level birth outcome measurements have suggested that total suspended particulates, particulate matter, PAHs, and their biologically active derivatives and proxy measures such as distance-weighted traffic density reduce BW and increase the risk of preterm birth and IUGR (Chen et al. 2002; Dejmek et al. 2000; Gouveia et al. 2004; Ha et al. 2001; Huynh et al. 2006; Lee BE et al. 2003; Leem et al. 2006; Liu et al. 2003; Perera et al. 1998, 2003; Sagiv et al. 2005; Wang et al. 1997; Wilhelm and Ritz 2003; Yang et al. 2003). Many of these studies have suffered from spatial and temporal exposure misclassification stemming from reliance on routine ambient air monitoring data to approximate personal exposure, multiple testing of mutually correlated air pollutants, and retrospective or cross-sectional exposure assessment that have prevented the investigators from disentangling the effects of individual pollutants (Šrám et al. 2005). In addition, only one study to date (Dejmek et al. 2000) has directly examined the association between airborne PAHs and IUGR (defined as BW below the 10th percentile), calling for replication.

We have previously demonstrated a significant association between prenatal PAH exposure and reduced BW and birth head circumference (BHC) in U.S. African Americans (Perera et al. 2003) and in Krakow Caucasians (Choi et al. 2006). It remains unclear from our analysis whether the reduced BW, including BW within the range appropriate for gestational age (AGA), reflects chronic, subacute, or acute IUGR or shortened gestation. Furthermore, BW has several well-known limitations as a constitutive fetal growth marker (Wilcox 2001).

IUGR is a broad definition that reflects failure of the fetus to reach its full growth potential (Lee PA et al. 2003; Smith et al. 1997). Although the term “intrauterine growth restriction” is often erroneously equated with small size for gestational age (SGA), IUGR diagnosis considers clinical and ultrasonography-based presentation of suboptimal growth, in addition to the fetal growth ratio below the 10th percentile (Smith et al. 1997). SGA is a population-based definition, which considers fetus as a case if the BW is less than the 10th percentile (Alexander et al. 1996) or 5th or 3rd percentile (Lee PA et al. 2003) for a given ethnic group, gestational age, and sex, or at least two standard deviations lower than the mean weight (Lee PA et al. 2003). SGA has been validated as predictor of neonatal morbidity and mortality and subsequent developmental delays (Lee PA et al. 2003; Thomas et al. 2000), including delayed neurodevelopment (Sizonenko et al. 2006; van Wassenaer 2005) and cardiovascular diseases, insulin resistance, and diabetes during adulthood (Barker 2002; Xu et al. 2006).

One of the disadvantages of SGA-based IUGR case detection is that newborns who fit the definition of SGA may not necessarily be growth restricted (if the constitutive growth potential has been reached), whereas acutely growth-restricted infants may have BW within the range of AGA (Lee PA et al. 2003). An alternative definition, fetal growth ratio (BW/mean BW for a given ethnic group, gestational age, and sex < 85%) (Frisbie et al. 1997), is also widely used in clinical diagnosis, management, and epidemiologic investigations of the underlying etiologies (Lee PA et al. 2003; Smith et al. 1997; Thomas et al. 2000). Several investigations have demonstrated that the ratio correlates with clinical and cytologic evidence of IUGR (Kramer et al. 1989, 1990a; Smith et al. 1997). A ratio < 85% has been associated with greater risk of stillbirth, hypoglycemia, hypocalcemia, various asphyxic symptoms, and neonatal death (Kramer et al. 1990b). Finally, recent reports suggest that infants born large for gestational age (LGA) are at greater risk for medical problems associated with metabolic syndromes, ranging from glucose intolerance and high blood pressure to dyslipidemia, type II diabetes, hypertension, and coronary heart disease in later life (Boney et al. 2005).

The phenomenon of IUGR can be further differentiated into symmetric and asymmetric subtypes based on recent observations that the severity of long-term morbidities differ according to body proportionality (Gluckman and Pinal 2003; Villar et al. 1990). Among SGA newborns, those with low ponderal index (i.e., “thin” at given height) are at significantly greater risk of perinatal asphyxia and extended hospitalization, compared with those with “appropriate” ponderal index (Villar et al. 1990). Cephalization index has been proposed as a screening tool for neurodevelopment IUGR subtype (Harel et al. 1985). Children 10 years of age who were born with larger head size relative to body weight (i.e., “brain spared”) scored significantly lower in neuro-developmental tests, IQ, and school performances (Leitner et al. 2007). Both ponderal index and cephalization index have been significantly correlated with severe IUGR and with poor neurodevelopmental outcomes (Bassan et al. 2005; Leitner et al. 2007) as well as with subsequent morbidity and mortality (Harel et al. 1985; Kramer et al. 1989, 1990a, 1990b; Leitner et al. 2007; Villar et al. 1990).

Of particular interest to the present analysis is a recent observation that the gestational age at IUGR onset appears to influence the type of IUGR (Hemachandra and Klebanoff 2006). IUGR cases whose period of onset was the third trimester have the lowest BW and ponderal index, and the IUGR cases whose onset were the first trimester have the highest BW and ponderal index (Hemachandra and Klebanoff 2006).

The primary goal of the present analysis is to examine the association between third-trimester exposure airborne to PAHs and IUGR. Considering that our previous analyses showed that prenatal PAH exposure was associated with reduced BW and BHC in New York City (Perera et al. 2003) and Krakow, Poland (Choi et al. 2006), we postulated that the PAH exposure is associated with two different markers of IUGR (SGA and fetal growth ratio < 85%, respectively). Furthermore, considering the gestational period of PAH monitoring, we tested a subhypothesis that high PAH exposure further contributes to the risk of severe IUGR (in terms of low ponderal index and/or high cephalization index). Lastly, we examined whether the same exposure also increases the risk of preterm delivery.

## Materials and Methods

### Study subjects

A complete description of the cohort and study design appears elsewhere (Perera et al. 2003; Rauh et al. 2004; Tonne et al. 2004). Briefly, African-American and Dominican women who reside in Washington Heights, Harlem, or the South Bronx in New York City were recruited through the local prenatal care clinics into a prospective cohort study (Perera et al. 2003). To reduce the potential for confounding, the target population was restricted to those with the following characteristics: age range of 18–35 years, non-cigarette smokers, nonusers of other tobacco products or illicit drugs; free of diabetes, hypertension, or known HIV; and initiated prenatal care by the 20th week of pregnancy. The institutional review board of the New York Presbyterian Medical Center approved the study, and informed consent was obtained from all study participants.

During the last trimester of pregnancy, a 45-min questionnaire on health, lifestyle, and exposure history was administered in the women’s homes. On completion of the pre-natal interview, the women were asked to wear small backpacks containing personal air monitors that operated for a consecutive 48-hr period (Perera et al. 2003). The personal air sampling pump operated continuously at 4L/min to simulate normal lung capacity. The particulate and semivolatile vapor and aerosol PAHs were collected on a precleaned quartz microfiber filter and polyurethane foam plug backup. The samples were analyzed for pyrene and eight carcinogenic PAHs: benz[*a*]anthracene, chrysene/isochrysene, benzo[*b*]fluoranthene, benzo[*k*]fluoranthene, BaP, indeno[*1,2,3-c*,*d*]pyrene, dibenz[*a*,*h*]-anthracene, and benzo[*g*,*h*,*i*]perylene. Of the 847 fully enrolled mother–newborn pairs, the sample was restricted to those participants with valid personal air monitoring data and a singleton birth, resulting in a sample size of 624. To reduce the potential for confounding by active maternal smoking in pregnancy, we eliminated from our analysis mothers or newborns whose plasma cotinine level was > 25 ng/mL (*n* = 8). Eight cases with implausible BW, length, or BHC for the reported gestational age (three times higher than the interquartile range) were treated as missing values. The present analysis included 224 African-American and 392 Dominican mother–newborn pairs (616 total). Because air monitoring did not occur until the third trimester, earlier preterm deliveries were not included. Birth outcome information was collected from mothers’ and infants’ medical records. The attending clinicians estimated the gestational age with the last menstrual period (LMP) or sonogram whenever the sonogram data were available. We collected the LMP date and sonogram-based expected date of delivery (both available for 70% of the sample) in our questionnaire, and validated the medical-record–based gestational age. Because identification of SGA depends on accurately determined gestational age, we previously demonstrated that the reported gestational age is reliable (Choi et al. 2006). Supporting the validity of the gestational age used in the present analysis, the mean difference in medical-record–reported versus our derived gestational age was 0.31 ± 1 week for LMP-based, 0.34 ± 2 for sonogram-based, and 0.62 ± 2 week for both LMP- plus sonogram-based gestational estimation methods.

### Outcomes

We identified cases of IUGR based on several definitions, recognizing the strengths and limitations of each approach. We first defined cases of SGA as BW ≤ 10th percentile at a given gestational age, within a plausible gestational age range, based on sex-specific U.S. singleton BW reference data (Alexander et al. 1999). This population-based definition of SGA represents the most widely used approach (Royal College of Obstetrician and Gynaecologists 2002). Newborns with BW at a given gestational age > 10th percentile and < 90th percentile were considered AGA, and those ≥ 90th percentile were classified as LGA, also based on the 1994–1996 U.S. singleton BW reference data (Alexander et al. 1999). In addition, we defined cases of SGA based on ethnicity- and sex-specific reference data (Alexander et al. 1999) because constitutive growth potential may vary across ethnic groups (Alexander et al. 1999; Frisbie et al. 1997).

Because the SGA definition might have misclassified constitutively small newborns as SGA, or acute IUGR newborns as AGA, we also calculated fetal growth ratio as the observed BW/mean BW at a given gestational age for each sex and ethnic group based on the 1994–1996 U.S. BW distribution (Frisbie et al. 1997). The ratio indicates the percentage underweight relative to the mean. Reduction in fetal growth ratio associated with PAH exposure was modeled using both dichotomous (< 85%) and continuous outcomes.

To examine the risk of disproportional IUGR, we used linear regression and multivariate logistic regression to test whether prenatal PAH exposure was associated with ponderal index (Villar et al. 1990) and cephalization index (Bassan et al. 2005; Leitner et al. 2007). Ponderal index [BW/(length)^3^ × 100], an indicator for thinness of the newborn, was calculated overall and for full-term births only. The cephalization index was calculated as the ratio of the BHC/BW × 10,000 (Bassan et al. 2005; Leitner et al. 2007).

### Exposure variable

The eight carcinogenic PAHs are significantly mutually correlated (Pearson’s correlation = 0.211–0.911; all *p*-values < 0.001). Because the relative fetotoxic potencies of the eight PAHs are unknown, we used the summed total of the eight carcinogenic PAHs (∑8 c-PAHs) as the main exposure variable, as in prior studies (Choi et al. 2006; Perera et al. 2003). The distributions of the summed PAHs as well as the eight individual ones deviated from normality (Kolmogorov–Smirnov test *p*-values < 0.001). The natural-log (ln)–transformed summed total c-PAHs conformed to the normal distribution.

### Potential confounders

We restricted our analysis to those newborns whose cord serum cotinine concentration was ≤ 25 ng/mL, to eliminate active maternal smokers from the sample. Independent risk factors and confounders identified from the literature were included in our initial models, including current marital status, maternal foreign birth, sex of the newborn, dietary PAH intake frequency, season of delivery (indicator variables, fall, winter, and spring), maternal education level, maternal prepregnancy body mass index (BMI), number of months of cigarette exposure in the home during pregnancy, maternal weight gain, and medical complications, including abruption of placenta and placenta previa. Maternal prepregnancy BMI was categorized as underweight (< 20), appropriate (20–24.9), overweight (25–29.9), or obese (≥ 30) (Cedergren 2007). To examine whether the lifestyle factors contribute to personal PAH exposure, mean personal exposure concentration of ∑8 c-PAHs was compared among the women according to the number of smokers at home, total months or daily number of hours of environmental tobacco smoke (ETS) exposure, and frequency of ingesting grilled, smoked, or barbecued food items. Variables were retained in the model when they resulted in > 10% change in the effect size of PAHs. To examine the robustness of our results, we assessed through regression diagnostics the possibility that influential values affected the estimated risk. We also examined whether the exclusion of cases because of missing information on confounders had a large effect on the estimated risks. We compared the included cases of SGA, fetal growth ratio < 85%, and preterm births with the excluded subjects in terms of BW, birth length, BHC, maternal weight gain, prepregnancy BMI, and total PAH exposure. There were no significant differences between the included and the excluded subjects in these variables in both ethnic groups. The prepregnancy BMI was significantly lower among the New York City Dominicans included in present analysis versus excluded subjects. We compared the risk between included and excluded using prepregnancy weight and height instead of BMI. The risk of prenatal PAH exposure was not significantly different in the groups, and remained unchanged when prepregnancy weight and maternal height were used. High dietary intake of PAHs during the current pregnancy was defined as having eaten smoked, grilled, or barbecued meat or fish at least twice a week (Choi et al. 2006).

### Model selection strategy

To assess relationships between prenatal PAH exposure and symmetric intrauterine growth restriction (IUGR), we developed separate logistic regression models for each ethnic group because previous results from this cohort showed significantly different PAH effects on the probability of reduced fetal growth in the two different ethnic groups (Perera et al. 2003). In the present logistic regression analysis, the initial model that included all potential confounders was reduced through hierarchical backward model selection strategy (Kleinbaum and Klein 2002). In particular, the –2 log likelihood value under the chi square distributions with α = 0.05 was compared for two models with and without the variable being considered for elimination. Adequacy of the model was determined using Nagelkerke’s coefficient of pseudo-*R*^2^, interpreted as the variation in outcome explained by the model. Maternal prepregnancy BMI and season of birth were retained in the models based on clinical relevance, regardless of the –2 log likelihood ratio results (Choi et al. 2006). Goodness of fit of the final model was examined through Hosmer–Lemeshow’s test. For the continuous outcome variables, similar criteria of modeling selection were used as in our previous analysis (Choi et al. 2006).

## Results

### Descriptive analysis

**Figure 1:**
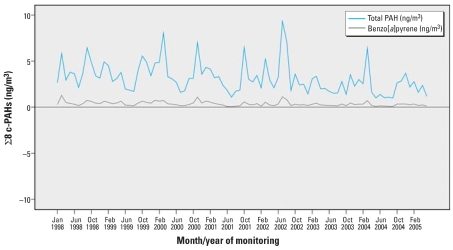
Monthly mean personal exposure to BaP and ∑8 c-PAHs (ng/m^3^).

The distribution of individual PAHs based on personal 48-hr monitoring is shown in Table 1. Individual ln-transformed PAHs and ∑8 c-PAHs across the monitoring period, January 1998 to February 2005, revealed no distinct secular trend (all *p*-values > 0.00001) (Figure 1). However, summer (June–August) levels of the individual and ∑8 c-PAHs were significantly lower than winter levels (December–February) (all *p*-values < 0.00001). The mean personal exposure to BaP over the entire monitoring period was 0.368 ± 0.464 ng/m^3^ (range, 0.043–1.298 ng/m^3^), consistent with the background ambient BaP level in New York City in 2002 (Pleil et al. 2004). Mean personal exposure to ∑8 c-PAHs averaged within ZIP code did not differ across the residential ZIP codes (analysis of variance, *p* > 0.8). Mean personal exposure levels also did not differ by location of prenatal care clinic (Manhattan vs. the South Bronx) (*p* = 0.38). Fifty-four percent of African-American and 77% of Dominican women reported absence of ETS exposure during the entire pregnancy. Among Dominicans, personal PAH exposure did not increase with the number of smokers at home, total months or the daily number of hours of ETS exposure, or frequency of ingesting grilled, smoked, or barbecued food items (*p* > 0.05). Among African Americans, although the reported frequency of PAH-laden food was not positively associated with the PAH exposure, a month of ETS exposure was associated with 3.9% increase in PAH exposure (*p* = 0.001), and a 1-hr increase in ETS exposure was associated with 4.3% increase in PAH exposure (*p* = 0.004). The reported hours of daily ETS exposure during the current pregnancy were significantly correlated with hours of daily exposure within the last 2 years in both ethnic groups (Pearson’s ρ = 0.90 and 0.80 among Dominicans and African Americans, respectively), suggesting temporal stability in PAH exposure.

The demographic and exposure characteristics of all mother–newborn pairs are shown in Table 2. Based on the overall U.S. BW reference (Alexander et al. 1996), more African-American newborns were identified as SGA (*n* = 33, 16%) and LGA (*n* = 27, 13%) than were Dominican newborns (SGA, *n* = 23, 6%; LGA, *n* = 22, 6%). The prevalence of newborns whose BW was < 85% relative to the mean weight was higher for the African Americans (*n* = 28, 13%) compared with the Dominicans (*n* = 39, 10%). The prevalence of preterm birth was 6% for African Americans (*n* = 14) and 3% for Dominicans (*n* = 11). This is small relative to national rates because, as explained above, we excluded high-risk pregnancies (maternal smokers and substance abusers) and women who delivered before the time of the air monitoring in the third trimester.

Based on the reported significant correlation between the degree of fetal growth ratio and disproportional fetal growth (measured in terms of ponderal index) (Kramer et al. 1989), we attempted to validate fetal growth ratio as a marker of IUGR in our cohort. Within both ethnic groups, reduction in fetal growth ratio was linearly associated with the BW, length, head circumference, cephalization index, and ponderal index. As shown in Table 3, mild to severe growth restriction (fetal growth ratio < 85%) was associated with progressively thinner but larger head to weight ratios. Although absolute head size (in centimeters) decreased with greater severity of growth restriction, head size relative to body weight increased, suggesting that brain growth might be supported while possibly restricting other organs.

With increasing exposure concentration of PAHs, the proportion of cases of SGA and fetal growth ratio < 85% increased (*p* < 0.05 for linear trend) in African Americans, but not in Dominicans (Table 4). Among the African Americans, although newborn cephalization index significantly increased with increasing exposure (*p* = 0.023), dose–response reduction in ponderal index was not observed (*p* = 0.176).

### Relationships between prenatal PAH exposure and measures of IUGR

#### Risk of proportional IUGR: SGA and fetal growth ratio < 85%

As shown in Table 5, controlling for maternal prepregnancy BMI, months of prenatal ETS exposure, parity, and winter delivery, a 1-ln-unit increase in prenatal PAH exposure was associated with a 2-fold increase in the likelihood of being born SGA among African Americans, regardless of whether SGA was based on the overall U.S. reference [odds ratio (OR) = 1.9; 95% confidence interval (CI), 1.1–3.5] or the ethnic-specific reference (OR = 2.4; 95% CI, 1.0–5.6). The risk of SGA was not significantly greater among underweight African-American women (*p*-value for interaction term = 0.16). Consistent with our prior findings, the risk of SGA was not elevated in Dominicans, either in the entire cohort or among those born full-term.

There was a significant inverse association between LGA and prenatal PAH exposure in African Americans (OR = 0.3; 95% CI, 0.1–0.8), but not for Dominicans (OR = 0.8; 95% CI, 0.4–1.5), controlling for maternal prepregnancy BMI, the number of months of prenatal ETS exposure, parity, and season of delivery.

Among African Americans but not Dominicans, a 1-ln-unit increase in PAH exposure was associated with a 5% reduction in fetal growth ratio (adjusted β = –4.865; *p* = 0.004). As shown in Table 5, a 1-ln-unit increase in prenatal PAH exposure was associated with a 2-fold increase in risk of having fetal growth ratio < 85% overall (95% CI = 1.0–3.6; *p* = 0.04), and among full-term births (95% CI = 1.1–4.2; *p* = 0.03).

#### Risk of disproportional IUGR: ponderal index and cephalization index

PAH exposure was not associated with a significant reduction in ponderal index in either African Americans (among all gestational ages: β = –0.042, or 4% decrease; *p* = 0.241; among full term: β = –0.052, or 5% decrease; *p* = 0.18) or Dominicans (among all gestational ages: β = 0.050, or 5% increase; *p* = 0.15; among full term: β = 0.046, or 5% increase; *p* = 0.15), after controlling for gestational age, sex, season of delivery, maternal prepregnancy BMI, gestational weight gain, parity, and months of prenatal ETS exposure.

On the other hand, a 1-ln-unit exposure increase was associated with a 0.04% increase in cephalization index in African Americans (β = 4.173; *p* = 0.023) and 0.02% decrease for Dominicans (β = –2.058; *p* = 0.019), controlling for gestational age, newborn sex, respective season of delivery, maternal education, BMI, gestational weight gain, parity, months of ETS exposure, and frequent dietary PAH intake during the current pregnancy.

### Relationship between prenatal PAH exposure and preterm delivery

We examined the association between prenatal PAH exposure and preterm delivery by first determining whether there was a linear dose–response relationship between PAH exposure and the length of gestational age, and whether the risk of preterm delivery increased with increasing exposure. Among African Americans, a 1-ln-unit increase in exposure was associated with a 2.5-day reduction in gestation (or 35% of a week) (*p* = 0.05), after controlling for maternal prepregnancy BMI, months of maternal exposure to ETS during the pregnancy, newborn sex, party, and season of delivery. A 1-ln-unit increase in prenatal PAH exposure was associated with a 5-fold greater risk of preterm delivery for African Americans (95% CI, 1.8–11.9; *p* = 0.001). Risk of preterm birth was not associated with prenatal PAH exposure among Dominicans (Table 6).

## Discussion

In this prospective cohort study, we found that among African-American newborns in New York City, a 1-ln-unit increase in prenatal PAH exposure was associated with approximately 2-fold greater risk of being born SGA or having a fetal growth ratio < 85%. There was a consistent doubling in risk of SGA whether the reference was total U.S. BW data or ethnicity-specific BW data. In addition, a 1-ln-unit increase in prenatal exposure was associated with a 0.04% increase in head size relative to BW (cephalization index) in African-American newborns, suggesting that growth restriction might induce a “brain-sparing” process at the developmental expense of other organs. Although our number of preterm births is small, our analysis indicates that a 1-ln-unit increase in PAH exposure increases the risk of preterm delivery among African Americans 5-fold (95% CI, 1.8–11.9).

By conducting a prospective cohort study incorporating personal air monitoring during pregnancy, we have addressed some of the traditional limitations in studies of the relation between air pollution and birth outcome, such as exposure misclassification and retrospective or cross-sectional exposure assessment. We have reduced potential confounding by restricting our cohort to healthy, nonsmoking, young women with no known risks of adverse birth outcomes. All pregnancies had successfully reached the third trimester, at which time the personal air monitoring was conducted.

A comparison between the two ethnic groups studied here revealed a higher occurrence of SGA in African-American newborns across the gestational age spectrum, including > 37 weeks. We therefore used both national (ethnic-combined) and ethnic-specific references because it is not known whether the different BW patterns are due to physiologic or pathologic causes (Kramer et al. 2006). However, recent investigations have demonstrated that SGA based on the overall (ethnic-combined) BW reference is the better predictor of neonatal mortality in U.S.-born blacks, suggesting that different BW patterns may not be physiologic (Joseph et al. 2005; Kramer et al. 2006). Our observation of significantly elevated risk of SGA is consistent with prior reports that SGA was associated with exposure to particulate matter with aerodynamic diameter ≤ 2.5 μm among California (USA) newborns (Parker et al. 2005) and with ambient PAH exposure among Czech Republic newborns (Dejmek et al. 2000). However, our estimated risk of SGA among African Americans is much higher than the risks reported by those researchers, despite the fact that mean PAH exposure in our cohort is about one-third of the mean exposure reported in the Czech Republic (Dejmek et al. 2000). As noted, SGA, one of the key proportional IUGR outcomes, has been associated with a significantly greater risk of delayed neurodevelopment (Sizonenko et al. 2006; van Wassenaer 2005), shorter stature, cardiovascular diseases, insulin resistance, and diabetes during adulthood (Barker 2002; Xu et al. 2006). Both SGA and LGA newborns have been shown to be at significantly greater risk of metabolic syndrome, including obesity, hypertension, dyslipidemia, glucose intolerance during childhood, and type II diabetes during adulthood (Boney et al. 2005). Considering the potential endocrine-disrupting properties of PAHs (Davis et al. 1993), we tested whether high PAH exposure increases the risk of being born LGA. Our present finding of a significantly higher likelihood of SGA and a significantly (66%) lower likelihood of being born LGA among New York City African Americans indicates that PAH effects are likely to be growth restricting, rather than growth promoting.

Our analysis of fetal growth ratio demonstrates that identification of IUGR cases should consider both population-normative (i.e., SGA) and maturity-based (i.e., fetal growth ratio) approaches. As a dichotomous category based on various percentile cutoff points, population-normative SGA has been criticized for including constitutively normal newborns (Lee PA et al. 2003; Royal College of Obstetrician and Gynaecologists 2002). In contrast, fetal growth ratio has been shown to correlate well with the clinical-based severity of IUGR (Kramer et al. 1989, 1990a). Our analysis also suggests that the percent deviation from the mean weight indicates the severity of IUGR. However, our estimated 5% reduction in fetal growth ratio per 1-ln-unit PAH exposure among African Americans warrants careful interpretation. Clinical severity and prognosis of 5% reduction would be greater for moderate to severe cases (fetal growth ratio < 80%) compared with the mild cases and noncases. Adverse clinical outcomes would likely be greater for infants with fetal growth ratio < 85% compared to those with higher fetal growth ratio.

IUGR is a broad category of suboptimal fetal growth, including both proportional and disproportional fetal growth. Thus, consideration of both statistically and clinically based definitions of IUGR is likely to improve case identification. Cephalization index, a validated marker of severe IUGR, significantly increased among African Americans with increasing pre-natal PAH exposure. Our finding is of concern given that children born with larger head circumference relative to body weight (i.e., “brain-spared”) score significantly lower in neurodevelopmental tests, IQ, and school performances (Leitner et al. 2007) and are at greater risk for subsequent morbidity and mortality (Harel et al. 1985; Kramer et al. 1989, 1990a, 1990b; Leitner et al. 2007; Villar et al. 1990). The finding of increased cephalization index and absence of risk for reduced ponderal index in African Americans warrants replication in a larger sample.

Our finding of increased risk among African Americans for preterm delivery suggests that PAHs might exert an array of biologic effects in the developing fetus. Specifically, symmetric IUGR appears to be independent of preterm delivery due to PAH exposure in our African-American sample: The risk of symmetric IUGR was significant both among all African-American newborns and among those born full term. Considering that the present cohort of pregnant women are at low risk of preterm delivery, our present findings of significant increase in preterm delivery additionally warrants replication in a carefully designed prospective cohort study.

None of the effects reported above in African Americans were seen in the Dominicans. We therefore examined possible sources of apparently greater susceptibility of African Americans. Although we did not ask about the specific dairy, fruit, fish, and vegetable consumption during the current pregnancy, at the follow-up interview conducted when the babies reached 2 years of age, 144 (68%) of Dominican and 120 (71%) of African-American mothers reported that they consumed fruits and vegetable regularly. Furthermore, 185 (87%) Dominican and 161 (96%) New York City African-American mothers reported that they took prenatal vitamins during the current pregnancy (Choi et al. 2006). Dietary exposure to PAHs was neither an independent risk factor nor a significant confounder for any of the birth outcomes. We have not seen convincing evidence in our data that micronutrient intake level or PAH dietary intake, alcohol use, or educational level (as a proxy for socioeconomic status) have confounded our results.

Consistent with our prior finding of no PAH effects on BW among Dominicans, we observed no significant increase in risk of SGA, reduced fetal growth ratio, increased cephalization index, or preterm delivery for Dominicans. The failure to find an adverse effect of PAH exposure on birth outcomes among Dominicans, despite the similarity in PAH exposure and socioeconomic status with African Americans in our cohort, is consistent with the paradoxical birth outcomes among Mexican immigrants living in the United States. Despite their high-risk demographic and socioeconomic profile, overall birth outcomes of Mexican-American newborns are comparable to those of U.S. non-Hispanic whites (Flores and Brotanek 2005). The apparent lack of association between airborne PAHs and adverse birth outcomes in our analysis might reflect healthful cultural practices among recent Dominican immigrants, including diets with higher nutritional quality, and greater social support. Possible protective effects of healthful cultural habits require careful further examination in the present cohort further studies, as do the specific factors underlying the greater vulnerability of the African Americans in our cohort.

## Conclusion

Because of the ubiquitous nature of PAH air pollutants produced by fossil fuel combustion, our observation that prenatal PAH exposure is associated with risk of SGA, preterm birth, and reduced fetal growth ratio and cephalization index among African Americans has implications for other urban populations and for environmental health policies.

## Figures and Tables

**Table 1:** ∑8 c-PAHs analyzed in prenatal air samples (ng/m^3^).

PAH	No.	Mean ± SD	Minimum	25th percentile	Median	75th percentile	Maximum
Benz[*a*]anthracene	612	0.259 ± 0.231	0.031	0.146	0.207	0.292	3.439
BaP	612	0.368 ± 0.464	0.021	0.147	0.248	0.446	6.203
Benzo[*b*]fluoranthene	604	0.543 ± 0.594	0.029	0.247	0.398	0.647	9.451
Benzo[*g*,*h*,*i*]perylene	613	0.985 ± 1.391	0.024	0.398	0.646	1.038	14.717
Benzo[*k*]fluoranthene	604	0.129 ± 0.125	0.014	0.046	0.097	0.164	1.594
Chrysene/isochrysene	612	0.335 ± 0.475	0.029	0.178	0.251	0.384	10.502
Dibenz[*a*,*h*]anthracene	612	0.054 ± 0.057	0.012	0.042	0.045	0.049	1.225
Indeno[*1*,*2*,*3-c*,*d*]pyrene	612	0.542 ± 0.656	0.024	0.222	0.374	0.600	7.409
∑8 c-PAHs	604	3.212 ± 3.400	0.271	1.545	2.299	3.662	36.466

**Table 2:** Demographic and exposure characteristics of mother–newborn pairs.^a^

	Dominican		African American		Total	
Characteristic	No. (%)	Mean ± SD	No. (%)	Mean ± SD	No. (%)	Mean ± SD
∑8 c-PAHs (ng/m^3^)	382	3.15 ± 3.42	222	3.32 ± 3.38	604	3.21 ± 3.40
Age (years)	389	25 ± 4	223	24 ± 4**	612	24 ± 4
Maternal weight gain during pregnancy^b^ (kg)						
Maternal BMI < 20	58 (16)	14.4 ± 6.0	31 (15)	12.4 ± 7.8	89 (16)	13.7 ± 6.7
Maternal BMI 20–24.9	167 (47)	14.1 ± 6.3	64 (31)	12.9 ± 5.6	231 (41)	13.8 ± 6.1
Maternal BMI 25–29.9	79 (22)	12.7 ± 5.9	46 (22)	13.1 ± 6.6	125 (22)	12.8 ± 6.2
Maternal BMI ≥ 30	53 (15)	11.9 ± 6.7	66 (32)	11.8 ± 7.6	119 (21)	11.9 ± 7.2
Total	357	13.5 ± 6.3	207	12.5 ± 6.8	564	13.1 ± 6.5
Maternal education						
< High school	139 (36.2)		75 (33.6)		214 (35.3)	
High school	155 (40.4)		90 (40.4)		245 (40.4)	
> High school	90 (23.4)		58 (26.0)		148 (24.4)	
Parity (yes)	280 (71.8)		182 (81.3)**		462 (75.2)	
Receive public assistance	149 (38.4)		104 (46.8)*		253 (41.5)	
Foreign-born mother	306 (78.3)**		13 (5.8)		319 (51.9)	
Receive Medicaid	360 (92.1)*		194 (87.0)		554 (90.2)	
High dietary PAH intake^c^	56 (15.3)		75 (34.2)**		131 (22.4)	
Any exposure during the current pregnancy						
Wine	47 (12.8)*		16 (7.3)		63 (10.8)	
Beer	71 (19.2)**		3 (1.4)		74 (12.6)	
Hard liquor	22 (6.0)		15 (7.0)		37 (6.4)	
Season of delivery						
Summer (Jun–Aug)	85 (21.9)		57 (26.3)		142 (23.4)	
Fall (Sep–Nov)	103 (26.5)		59 (27.2)		162 (26.7)	
Winter (Dec–Feb)	87 (22.4)		49 (22.6)		136 (22.4)	
Spring (Mar–May)	114 (29.3)		52 (24.0)		166 (27.4)	

a Numbers vary because of missing information for some cases.

b Weight gained during the current pregnancy was stratified according to prepregnancy BMI categories.

c Reported eating at least one smoked, grilled, or barbecued food more than twice a week.

* *p* < 0.05,

** *p* < 0.01, based on *t*-test and chi-square analysis.

**Table 3:** Newborn anthropometric indicators according to severity of fetal growth ratio.^a^

	BW (g)		Birth length (cm)		BHC (cm)		Ponderal index (g/cm^3^ × 100)		Cephalization index (cm/g × 10^4^)	
Mother–infant pair group	No.	Mean ± SD	No.	Mean ± SD	No.	Mean ± SD	No.	Mean ± SD	No.	Mean ± SD
African American*										
Noncase	177	3,417 ± 432	174	51 ± 2	171	34 ± 1	174	2.55 ± 0.28	171	101.04 ± 10.44
Mild	18	2,619 ± 178	17	48 ± 2	17	32 ± 2	17	2.39 ± 0.30	17	122.44 ± 6.27
Moderate	2	2,655 ± 57	2	51 ± 1	2	33 ± 1	2	2.00 ± 0.12	2	122.41 ± 0.06
Severe	8	2,031 ± 341	8	45 ± 2	8	31 ± 2	8	2.21 ± 0.20	8	154.91 ± 29.01
Dominican*										
Noncase	302	3,511 ± 389	283	51 ± 2	275	35 ± 1	283	2.64 ± 0.41	275	99.19 ± 9.00
Mild	17	2,870 ± 105	14	50 ± 2	11	33 ± 1	14	2.36 ± 0.24	11	114.41 ± 5.42
Moderate	14	2,588 ± 239	2	48 ± 3	11	33 ± 1	13	2.36 ± 0.27	11	126.06 ± 7.67
Severe	7	2,449 ± 213	6	48 ± 2	6	32 ± 1	6	2.25 ± 0.33	6	131.37 ± 11.87
Total*										
Noncase	479	3,477 ± 407	457	51 ± 2	446	34 ± 1	457	2.61 ± 0.37	446	99.90 ± 9.61
Mild	35	2,741 ± 193	31	49 ± 2	28	32 ± 2	31	2.38 ± 0.27	28	119.29 ± 7.08
Moderate	16	2,596 ± 224	15	48 ± 3	13	33 ± 1	15	2.31 ± 0.28	13	125.50 ± 7.13
Severe	15	2,226 ± 352	14	46 ± 3	14	31 ± 1	14	2.23 ± 0.25	14	144.82 ± 25.56

a Severity was defined as noncase (fetal growth ratio ≥ 85), mild (fetal growth ratio = 80–84.99), moderate (fetal growth ratio = 75–79.99), and severe (fetal growth ratio < 75) (Kramer et al. 1989).

* Test of linear trend for all presented outcomes with increasing severity, *p* < 0.001.

**Table 4:** SGA, fetal growth ratio, ponderal index, and cephalization index with increasing PAH exposure concentration.

	Size for gestational age		Fetal growth ratio		Ponderal index	Cephalization index
	AGA (reference)	SGA	≥ 85% (reference)	< 85%		
African American						
∑8 c-PAHs (ng/m^3^)						
≤ 1.55	37 (21.0)	3 (9.1)	41 (21.1)	4 (14.3)	2.48 ± 0.30	104.17 ± 22.52
1.56–2.30	47 (26.7)	4 (12.1)	49 (25.3)	4 (14.3)	2.63 ± 0.31	101.20 ± 10.95
2.31–3.66	50 (28.4)	12 (36.4)	59 (30.4)	6 (21.4)	2.52 ± 0.28	102.89 ± 13.04
≥ 3.67	42 (23.9)	14 (42.4)*	45 (23.2)	14 (50.0)*	2.44 ± 0.26	111.94 ± 17.64**
Dominican						
∑8 c-PAHs (ng/m^3^)						
≤ 1.55	94 (27.9)	4 (17.4)	98 (28.5)	8 (21.1)	2.54 ± 0.29	103.04 ± 11.14
1.56–2.30	85 (25.2)	8 (34.8)	85 (24.7)	13 (34.2)	2.68 ± 0.64	99.82 ± 10.19
2.31–3.66	74 (22.0)	8 (34.8)	76 (22.1)	10 (26.3)	2.63 ± 0.32	101.37 ± 11.72
≥ 3.67	84 (24.9)	3 (13.0)	85 (24.7)	7 (18.4)	2.61 ± 0.28	101.12 ± 12.12

Values are no. (%) or mean ± SD.

* *p* < 0.05 and

** *p* < 0.01 for linear trend in outcome with increasing exposure.

**Table 5:** Risk of SGA and fetal growth ratio.

	SGA^a^				Fetal growth ratio < 85%^b^			
	Ethnicity-combined U.S. BW percentile^c^		Ethnicity- and sex-specific U.S. BW percentile^d^		All births^e^		Full term only	
	OR (95% CI)	*p*-Value	OR (95% CI)	*p*-Value	OR (95% CI)	*p*-Value	OR (95% CI)	*p*-Value
African American	*n* = 201		*n* = 201		*n* = 205^b^		*n* = 194	
Ln-unit PAH exposure	1.94 (1.09–3.47)	0.03	2.43 (1.05–5.62)	0.04	1.93 (1.04–3.56)	0.04	2.15 (1.09–4.25)	0.03
Dominican	*n* = 336		*n* = 332		*n* = 349		*n* = 341	
Ln-unit PAH exposure	0.82 (0.44–1.51)	0.53	0.87 (0.51–1.47)	0.60	0.82 (0.51–1.33)	0.42	0.80 (0.48–1.33)	0.40

a SGA model controls for maternal BMI, months of gestational ETS exposure, parity, and winter delivery.

b Fetal growth ratio < 85% model controlled for BMI, gestational weight gain, months of gestational ETS exposure, and parity.

c Thirty-three cases and 168 noncases for African Americans; 20 cases and 316 noncases for Dominicans.

d Fourteen cases and 187 noncases for African Americans; 27 cases and 305 noncases for Dominicans.

e Among all births, the number of cases with fetal growth ratio < 85% was 26 among African Americans and 34 among Dominicans. When sample was restricted to full-term birth only, there were 23 cases versus 171 noncases in African Americans, and 30 cases versus 311 noncases among Dominicans.

**Table 6:** Risk of shortened gestational age and preterm delivery.^a^

	Gestational age		Preterm delivery^b^	
	β (95% CI)	*p*-Value	OR (95% CI)	*p*-Value
African American (*R*^2^ = 0.039)	(*n* = 201)		(*n* = 205)	
Ln-unit PAH exposure	–0.354 (–0.714 to 0.006)	0.053	4.676 (1.839 to 11.886)	0.001
Dominican (*R*^2^ = 0.027)	(*n* = 335)		(*n* = 351)	
Ln-unit PAH exposure	–0.006 (–0.190 to 0.178)	0.952	0.523 (0.182 to 1.504)	0.229

a Model controlled for maternal prepregnancy BMI, sex, parity, delivery season, and months of gestational ETS exposure.

b Number of preterm cases (*n* = 12 for African Americans; *n* = 8 for Dominicans) was reduced because of missing data in independent predictors.
